# Three-dimensional evaluation of soft tissue contour changes after premolar extraction with and without alveolar ridge preservation: A prospective observational study

**DOI:** 10.1371/journal.pone.0342338

**Published:** 2026-02-27

**Authors:** Yun-Jeong Kim, Mi Young Lee, Jae-Eun Chung, Jang-Hyun Kim, Ji-Man Park, Ki-Tae Koo, Young Ku

**Affiliations:** 1 Department of Periodontology, Seoul National University Gwanak Dental Hospital, Seoul, Korea; 2 Department of Periodontology and Dental Research Institute, School of Dentistry, Seoul National University, Seoul, Korea; 3 Department of Orthodontics, Seoul National University Gwanak Dental Hospital, Seoul National University School of Dentistry, Seoul, Korea; 4 Department of Prosthodontics and Dental Research Institute, School of Dentistry, Seoul National University, Seoul, Korea; International Medical University, MALAYSIA

## Abstract

**Background:**

This prospective observational study evaluated three-dimensional soft tissue contour changes over a 90-day healing period following bilateral premolar extractions, with and without alveolar ridge preservation (ARP), using intraoral scanning and 3D digital analysis.

**Methods:**

Eighteen orthodontic patients requiring bilateral premolar extractions were enrolled. In each patient, the extraction socket with relatively lower buccal bone height was assigned to alveolar ridge preservation (ARP), while the contralateral site was left to heal spontaneously. Intraoral scans were obtained immediately before extraction and at 30 days and 90 days post-extraction. Linear horizontal soft tissue changes were measured at 2 mm (H2) and 4 mm (H4) below the gingival margin, while vertical changes were assessed at the central aspect of the extraction socket. Surface displacement was analyzed using root mean square (RMS) distance, and volumetric changes were quantified using region-of-interest segmentation.

**Results:**

Significant time-dependent changes in soft tissue contours were observed (p < .001), with greater horizontal reduction at H2 than H4, and more pronounced buccal changes (p < .05). At day 90, mean horizontal reduction at H2 buccal was 1.84 ± 0.69 mm (ARP) and 1.72 ± 0.68 mm (control); corresponding H2 lingual reductions were 1.34 ± 0.89 mm and 1.16 ± 0.29 mm. Vertical reduction peaked at 30 days with partial recovery by day 90. RMS-based displacement was greater buccally. Volumetric reduction was approximately 17% in both groups. No statistically significant differences were found between groups.

**Conclusions:**

Intraoral scanning combined with digital superimposition proved effective for evaluating soft tissue changes after extraction. While ARP remains a valuable approach for alveolar bone preservation, its additional impact on soft tissue contour was limited in sites with favorable baseline conditions.

**Trial registration:**

Clinical Research Information Service (CRIS), KCT0010923. Registered 19 August 2025. Retrospectively registered.

## Introduction

Alveolar ridge preservation (ARP) is a widely used procedure that reduces physiological resorption after tooth extraction, thereby facilitating tooth replacement and restoration [1]. Immediate grafting of extraction sockets is believed to serve as a scaffold for bone formation and ridge contour reconstruction, and is known to minimize dimensional loss, a common consequence of both soft and hard tissue remodeling [2,3]. Numerous studies support the superiority of ARP compared to spontaneous healing in attenuating the horizontal and vertical reduction of post-extraction ridges for periodontally damaged sockets as well as for intact sockets [4–6]. Although volumetric or linear bone resorption was significantly less in the case of ARP, interestingly, there were cases in which no significant difference was observed in terms of soft tissue contour change [7].

As in Thoma’s study [8], it is known that the effect of ARP can be confirmed by comparing and observing changes in the appearance of soft tissues with time after tooth extraction. However, to our knowledge, there have been few studies comparing natural healing and ridge preservation in symmetrically positioned extraction sockets within the same oral cavity. In clinical practice, conducting a split-mouth controlled study under comparable conditions is challenging because patients seldom require the simultaneous extraction of contralateral teeth in the same arch. [9–11]. Nevertheless, the limited number of published split-mouth studies have demonstrated that ARP can partially reduce vertical height loss and horizontal ridge dimension loss [12].

Although the premolar region is the primary indication for immediate implant placement, it is also frequently selected as a target site for alveolar ridge preservation, where several studies have demonstrated a statistically significant reduction in soft tissue volume loss with the application of grafting materials [13,14]. Based on this, in patients who require bilateral premolar extraction for orthodontic reasons, a study design that performs ARP on only one side and compares it with a control group may be considered. In fact, a small pilot study of orthodontic patients reported that areas treated with Guided Bone Regeneration (GBR) experienced less soft tissue atrophy, such as gingival recessions, compared to their counterparts [15]. Studies comparing soft tissue changes after orthodontic extraction, using a split-mouth design regardless of whether a bone graft is used, are more common [16].

A three-dimensional (3D) digital impression obtained using an intraoral scanner is gaining attention as a novel method that can alleviate patient discomfort associated with traditional impression-taking techniques and reduce errors in soft tissue volume measurements caused by pressure during the model-making process [17,18]. Therefore, to accurately implement the soft tissue contour, 3D images obtained through direct intraoral scans of patients were superimposed to investigate changes in soft tissue appearance following tooth extraction and the healing process.

The purpose of this clinical investigation was to compare the changes in soft tissue contours over 90 days following the extraction of bilateral premolars in orthodontic patients, with or without the application of alveolar ridge preservation.

## Materials and methods

### Study design and ethics

This study was designed as a prospective, split-mouth, comparative observational study conducted at Seoul National University Gwanak Dental Hospital, in accordance with the Declaration of Helsinki. Participants were prospectively recruited between December 6, 2019 and June 22, 2022.

**Fig 1:**
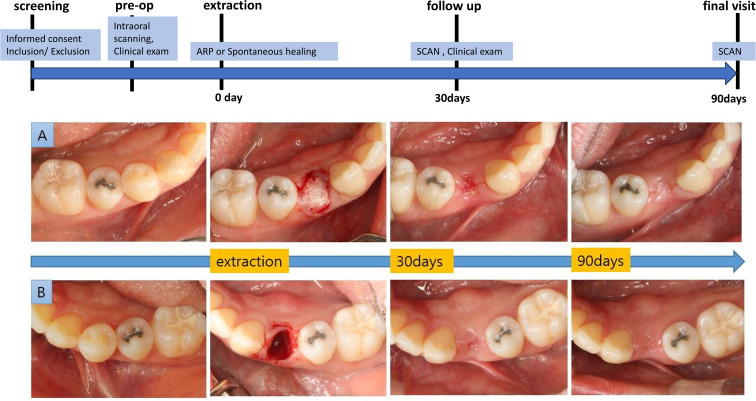
Experimental outline. **(A)** Site treated with alveolar ridge preservation (ARP) using a xenogeneic bone graft and collagen membrane. **(B)** The contralateral site was left to heal spontaneously without grafting.

The study was approved by the Institutional Review Board of Seoul National University Dental Hospital (IRB No. CRI19010/ IRB116/08–19; IRB334/08–20; and CRI21016/ IRB168/08–21). The entire study period was conducted under continuous ethical oversight, and no participant recruitment or study-related intervention occurred during the brief administrative interval between approvals. Written informed consent was obtained from all participants prior to enrollment. The study was retrospectively registered in the Clinical Research Information Service (CRIS, KCT0010923) to enhance transparency, as this observational study was initiated before prospective trial registration was considered mandatory. No protocol deviations occurred, and all procedures followed the prespecified IRB-approved protocol. All clinicians and examiners were calibrated before performing procedures and analyses. The study adhered to the STROBE guidelines for reporting observational research. The overall experimental design and timeline are illustrated in Fig 1.

### Patient selection and sample size calculation

Between December 2019 and June 2022, 18 orthodontic patients (≥18 years) requiring bilateral mandibular premolar extractions were enrolled. Patients with systemic contraindications (e.g., uncontrolled diabetes, pregnancy, smoking >10 cigarettes/day, immunosuppressive therapy, or drug-induced gingival overgrowth), allergies to graft materials, or compromised adjacent teeth (mobility, probing depth >4 mm, or missing) were excluded.

Sample size estimation was based on a previous split-mouth study involving 15 patients [12]. With a Type I error of 0.05 and 90% power, the required sample size was calculated as 18 patients. To account for potential dropouts, 20 participants were planned for recruitment, and 18 completed the study and were included in the analysis.

### Surgical procedures

At extraction, the socket with relatively lower buccal bone height was assigned to alveolar ridge preservation (ARP), while the contralateral site was left to heal spontaneously. Extractions were performed flaplessly with minimal trauma under local anesthesia by a single surgeon (YJK). ARP sockets were grafted with 100 mg of deproteinized bovine bone mineral with 10% collagen (Bio-Oss® Collagen; Geistlich Pharma AG, Switzerland) and covered with a double layer of collagen membrane (Bio-Gide®; Geistlich Pharma AG, Switzerland), stabilized by hidden-X [19] and Vicryl® 5−0 sutures. Control sockets were sutured without grafting. Standard postoperative care included antibiotics and analgesics for 5 days and chlorhexidine rinses until suture removal at 2 weeks. Orthodontic movement was postponed for 90 days.

### Intraoral scanning and measurements

**Fig 2:**
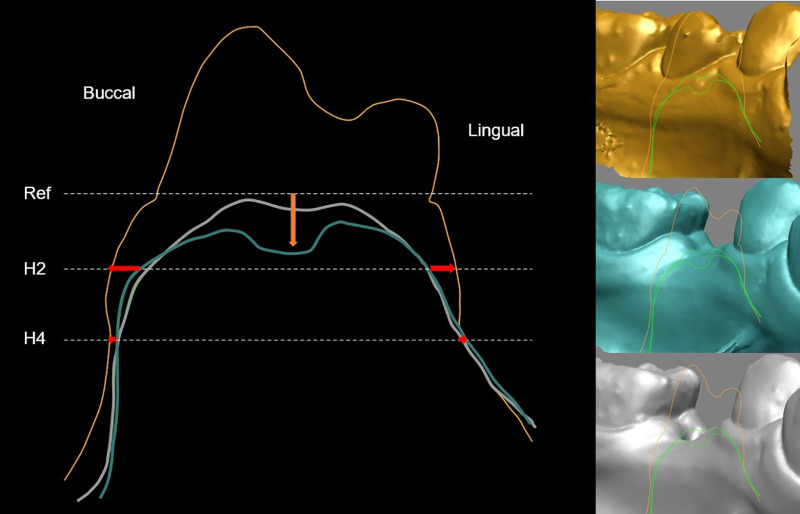
Cross-sectional view of the region of interest. Superimposed intraoral scan-derived STL models obtained at three time points: immediately before tooth extraction (yellow), 30 days post-extraction (blue), and 90 days post-extraction (white). Models were registered based on adjacent teeth. A reference line was drawn through the buccal and lingual gingival margins. Horizontal dimensional changes (HW; red arrows) were measured at 2 mm (H2) and 4 mm (H4) apical to the reference line. Vertical dimensional changes (orange arrow) were quantified perpendicular to the reference line at the center of the ridge.

Scans (i500; Medit, Korea) were obtained immediately before extraction and at 30 days and 90 days after extraction. STL datasets were superimposed with adjacent teeth as references (Geomagic Verify 64; 3D Systems, USA). Intraoral scans were acquired using an i500 scanner (Medit, Korea) with the corresponding scanner software (Medit Link; Medit, Korea) available during the data acquisition period. The scanner software does not provide user-adjustable options for mesh smoothing or decimation, and no additional mesh smoothing or decimation was applied to the exported STL files using external software. Surface registration was performed exclusively using adjacent teeth as reference structures, and gingival surfaces were excluded from the registration process. Linear measurements were made at 2 mm and 4 mm below the gingival margin (H2, H4) for horizontal width and at the center of the socket for vertical height (Rapidform 2006; INUS Technology, Korea) (Fig 2) [8,20]. For 3D analyses, volumetric and surface changes were calculated using region-of-interest segmentation and RMS distance (Geomagic Verify 64; 3D Systems, USA). Based on previously described methodologies [21,22], the region-of-interest (ROI) was defined by vertical reference lines drawn through the mesial and distal papillae of adjacent teeth, and by horizontal planes positioned 1 mm and 3 mm apical to the gingival margin. To evaluate surface deviations between baseline and follow-up scans, the root mean square (RMS) distance was calculated, providing an index of average point-to-point discrepancy within the ROI. Furthermore, buccal and lingual surfaces within each ROI were segmented and reconstructed into polygonal meshes, from which volumetric (VOL) differences were computed. All measurements were repeated twice by a calibrated examiner (JHK), and mean values were analyzed. Surface registration was performed using the “Max Average Deviation = Auto” setting in Geomagic Verify 64 (3D Systems, USA). Under this mode, the software applies an internally defined automatic convergence tolerance during the iterative closest point (ICP) registration process, and no manually specified tolerance threshold was applied. Intra-examiner reliability was assessed using intraclass correlation coefficients (ICC). A subset of sites was randomly selected and remeasured by the same examiner at a different time point. ICC values were calculated for all linear, volumetric, and RMS measurements using a two-way mixed-effects model with absolute agreement and single measures (ICC (3,1)).

### Statistical analysis

Statistical analyses were performed in SPSS Statistics 23 (IBM). The effects of treatment (ARP vs control), time (baseline, 30, 90 days), and their interaction were evaluated using repeated-measures ANOVA (Greenhouse–Geisser correction when sphericity was violated). Post hoc pairwise comparisons between groups at each time point were performed with Bonferroni adjustment (α/3 = 0.017). Within-subject paired comparisons (e.g., H2 vs. H4; buccal vs. lingual) were tested using Wilcoxon signed-rank tests, with multiplicity adjusted by the Bonferroni/Holm method. Statistical significance was set at p < 0.05. For repeated-measures ANOVA, effect sizes were calculated using partial eta squared (partial η²), and Greenhouse–Geisser corrections were applied when the assumption of sphericity was violated.

## Results

A total of 18 patients (mean age, 31.8 years; range, 20–65 years) were enrolled in this study. Bilateral premolar extractions were performed, resulting in a total of 36 extraction sites. Ridge preservation was conducted on one side, while the contralateral site healed spontaneously.

Repeated-measures ANOVA revealed significant time-dependent changes in both horizontal and vertical soft tissue dimensions following tooth extraction (*p <* .001). Horizontal width changes at both the buccal and lingual aspects were evaluated at two reference levels (H2: 2 mm and H4: 4 mm apical to the gingival margin). Significant reduction was observed over time at all sites (*p* < .001). Dimensional changes were more pronounced at the shallower (H2) level compared to H4 (*p <* .05).

**Fig 3:**
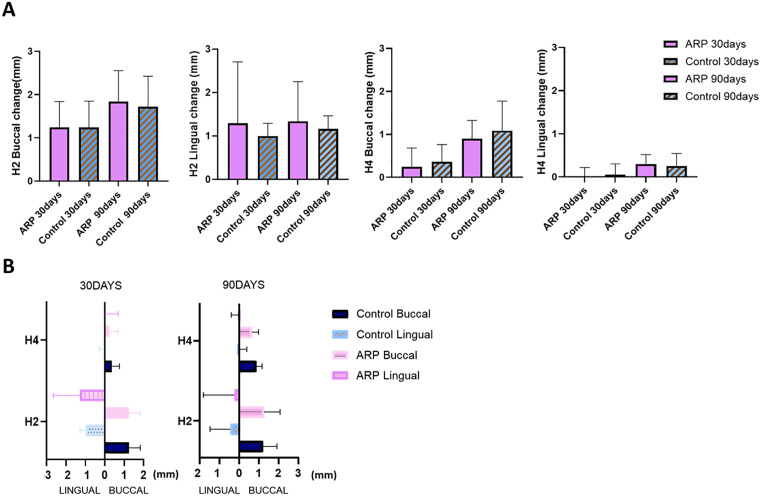
Linear changes in ridge contour over time. **(A)** Time-dependent horizontal changes at buccal and lingual aspects measured at H2 and H4. **(B)** Comparison of linear changes across positions at 30 and 90 days post-extraction. H2: horizontal width 2 mm apical to the reference line; H4: horizontal width 4 mm apical to the reference line; ARP: alveolar ridge preservation.

At day 90, the mean horizontal reduction at H2 buccal was 1.84 ± 0.69 mm in the ARP group and 1.72 ± 0.68 mm in the spontaneous healing group, while at H2 lingual it was 1.34 ± 0.89 mm and 1.16 ± 0.29 mm, respectively. Although no significant buccal–lingual difference was detected at H2 on day 30, significant differences emerged at H4 at both 30 and 90 days. At H4 buccal on day 90, mean reductions were 0.90 ± 0.42 mm (ARP) and 1.09 ± 0.67 mm (control), compared to 0.30 ± 0.21 mm and 0.25 ± 0.29 mm on the lingual side, respectively (Fig 3).

**Fig 4:**
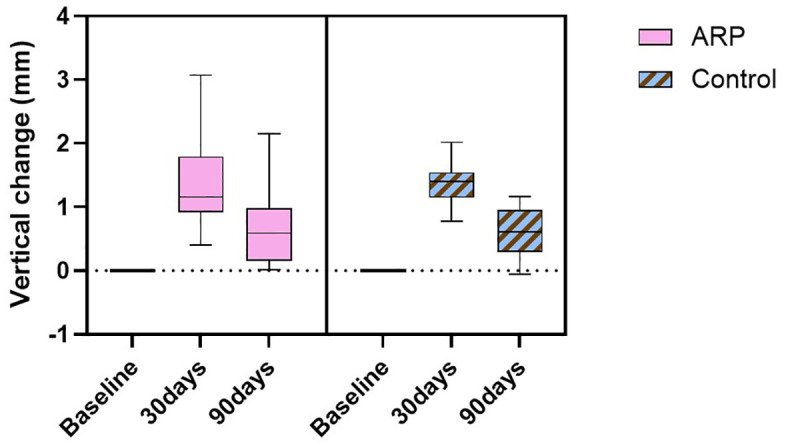
Vertical linear changes in ridge contour. Vertical dimensional changes were measured at the center of the extraction socket at baseline, 30 days, and 90 days post-extraction. ARP: alveolar ridge preservation.

Vertical dimensional changes at the center of the extraction socket exhibited a distinct temporal pattern. A marked reduction in ridge height was observed at 30 days post-extraction, followed by partial recovery by day 90 (Fig 4). Specifically, the mean vertical decrease at day 30 was 1.31 ± 0.67 mm (ARP) and 1.35 ± 0.3 mm (control), with corresponding values at day 90 of 0.67 ± 0.54 mm and 0.61 ± 0.36 mm, respectively.

**Fig 5:**
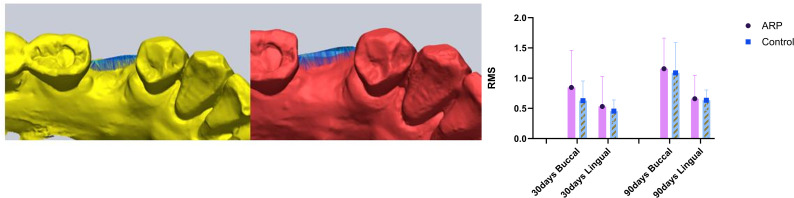
Mean distance between surfaces (RMS). Mean distance (root mean square, RMS) between superimposed surface models over time, quantifying volumetric soft tissue changes. ARP: alveolar ridge preservation.

RMS-based surface displacement analysis further corroborated these trends. At day 90, buccal RMS values were 1.16 ± 0.49 mm (ARP) and 1.09 ± 0.48 mm (control), while lingual values were 0.66 ± 0.38 mm and 0.63 ± 0.17 mm, respectively, indicating consistently greater displacement at the buccal aspect (*p <* .05) (Fig 5).

**Fig 6:**
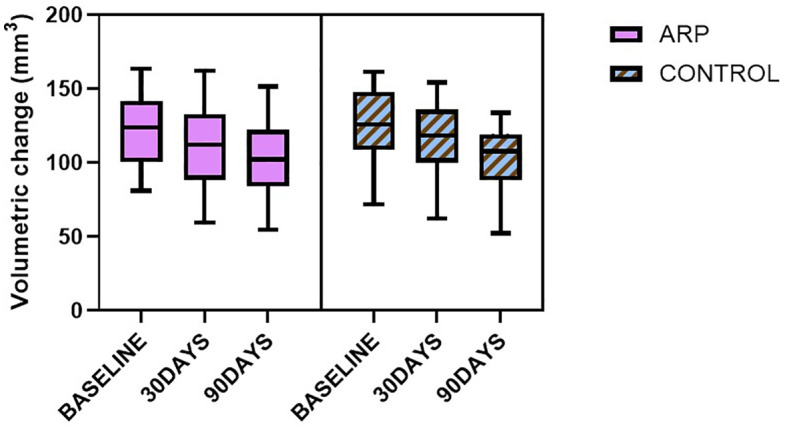
Volumetric changes in the extraction site. Three-dimensional volumetric changes were calculated by extracting regions of interest (ROIs) defined by consistent buccal and lingual borders and measuring the enclosed polygonal volume. ARP: alveolar ridge preservation.

Complementary volumetric analysis based on 3D model superimposition and region-of-interest (ROI) segmentation also demonstrated a significant reduction over time. In the ARP group, mean alveolar volume decreased from 121.62 mm³ pre-extraction to 100.99 mm³ at day 90 (a reduction of approximately 16.96%), while in the control group it decreased from 123.99 mm³ to 102.91 mm³ (approximately 16.99% reduction) (Fig 6). No significant intergroup differences were observed. Repeated-measures ANOVA showed significant main effects of time on both H4 buccal horizontal width (Greenhouse–Geisser corrected: F (1.75, 59.51) = 76.66, p < 0.001, partial η² = 0.69) and volumetric change (F (1.67, 56.75) = 111.69, p < 0.001, partial η² = 0.77). No significant interaction between time and treatment was observed for either outcome (H4 buccal: F (1.75, 59.51) = 0.67, p = 0.498, partial η² = 0.02; volume: F (1.67, 56.75) = 0.23, p = 0.759, partial η² = 0.01).

All measurements were derived from superimposed intraoral scan datasets. Horizontal and vertical linear dimensions were obtained at predefined reference levels, while surface displacement and volumetric changes were calculated based on 3D ROI segmentation. Each measurement was performed in duplicate, and mean values were used for the final analysis. Intra-examiner reliability was excellent across all measurements. Intraclass correlation coefficients ranged from 0.991 to 1.000 for linear measurements, were 1.000 for volumetric measurements, and ranged from 0.99 to 1.00 for RMS measurements. A summary of all measurement data is provided in Table 1.

**Table 1:** Changes in ridge contour between baseline and 30, 90 days post-extraction based on intraoral scanned data measurements (mean ± SD).

Variable			ARP		Control	
			Mean	SD	Mean	SD
**H2** **change** **(mm)**	**Buccal**	30days	1.24^a^	0.58	1.24^a^	0.59
		90days	1.84^ab^	0.69	1.72^ab^	0.68
		*p-value*	<0.001		<0.001	
	**Lingual**	30days	1.29^a^	1.37	1^a^	0.28
		90days	1.34^ab^	0.89	1.16^ab^	0.29
		*p-value*	<0.001		<0.001	
**H4** **change** **(mm)**	**Buccal**	30days	0.24^ab^	0.43	0.37^ab^	0.38
		90days	0.9^ab^	0.42	1.09^ab^	0.67
		*p-value*	<0.001		<0.001	
	**Lingual**	30days	0^ab^	0.69	0.05^ab^	0.24
		90days	0.3^ab^	0.21	0.25^ab^	0.29
		*p-value*	<0.001		<0.001	
**RMS**	**Buccal**	30days	0.85^b^	0.60	0.63	0.32
		90days	1.16^b^	0.49	1.09^b^	0.48
		*p-value*	<0.001		<0.001	
	**Lingual**	30days	0.53^b^	0.48	0.45	0.18
		90days	0.66^b^	0.38	0.63^b^	0.17
		*p-value*	<0.001		<0.001	
**VOL** **(mm**^**3**^)		Baseline	121.62	25.07	123.99	26.38
		30days	110.09	26.84	113.81	23.99
		90days	100.99	24.59	102.91	22.51
		*p-value*	<0.001		<0.001	
**Vertical change** **(mm)**		30days	1.31	0.67	1.35	0.3
		90days	0.67	0.54	0.61	0.36
		*p-value*	<0.001		<0.001	
**H2 Total length (mm)**		Baseline	9.69	0.67	9.82	0.76
		30days	7.16	1.94	7.58	1.04
		90days	6.51	1.71	6.93	1.07
		*p-value*	<0.001		<0.001	
**H4 Total length (mm)**		Baseline	10.26	0.87	10.57	1.09
		30days	10.02	1.05	10.15	1.35
		90days	9.06	1.12	9.24	1.45
		*p-value*	<0.001		<0.001	

Significant differences between H2 and H4 within the same group are indicated by superscript a (p < .05). Significant differences between Buccal and Lingual sides at the same level and time point are indicated by superscript b (p < .05).

H2: horizontal width 2 mm inferior to the reference line; H4: horizontal width 4 mm inferior to the reference line; ARP: alveolar ridge preservation; RMS: root mean square.

## Discussion

This prospective comparative observational study investigated soft tissue contour changes following tooth extraction using intraoral scan-based linear and volumetric analyses. Through a split-mouth design comparing alveolar ridge preservation (ARP) with spontaneous healing, the study provided high-resolution, non-invasive insights into early soft tissue remodeling over a 90-day period.

The present results demonstrated significant time-dependent alterations in both horizontal and vertical soft tissue dimensions. In accordance with prior findings [4,23,24], horizontal reduction of the ridge was more pronounced than vertical reduction, with greater dimensional loss observed at the buccal aspect compared to the lingual side. This pattern reflects the well-established healing dynamics of extraction sockets, where the buccal bone—predominantly composed of bundle bone—undergoes substantial vertical resorption during early healing [24]. The vertical resorption of the buccal bone is accompanied by horizontal atrophy of the alveolar ridge, driven by osteoclastic activity on the outer surfaces of both buccal and lingual walls [24]. These biological processes contribute to the characteristic buccal predominance of dimensional changes observed in the present and previous investigations.

Surface deviation analysis using root mean square (RMS) distance measurements further supported these findings, revealing progressive morphological changes over time. Greater deviation values were consistently observed at the buccal aspect, indicating more substantial surface displacement in this region. Complementary volumetric analysis, based on 3D region-of-interest (ROI) segmentation, likewise demonstrated a significant reduction in ridge volume over time, with no significant differences between groups.

Vertical reduction at the center of the extraction site was pronounced at day 30, with partial recovery by day 90. Both the ARP and spontaneous healing groups underwent standardized soft tissue management with identical suturing protocols. In both groups, secondary healing at the center initially resulted in transient depression, which progressively resolved as tissue maturation restored ridge contour stability by day 90.

Notably, despite these time- and site-dependent remodeling patterns, no statistically significant differences were observed between the ARP and control groups in either linear or volumetric soft tissue measurements. This contrasts with the majority of previous studies [2,7], which have reported beneficial effects of ARP on preserving both soft and hard tissue dimensions. The absence of significant group differences in this study may reflect the favorable baseline conditions of the extraction sites, including intact alveolar walls and healthy adjacent bone. Under such circumstances, spontaneous healing alone may maintain soft tissue stability, limiting the additional benefit of ARP. These results highlight the potential influence of baseline site characteristics on the magnitude of clinical outcomes achieved with ridge preservation, as previous studies [1,25] have suggested that the initial socket condition may modulate the extent of treatment benefit.

From a clinical standpoint, these results suggest that while ARP remains an effective approach for preserving alveolar ridge volume, its additional impact on soft tissue contour stability may be limited in sites with intact alveolar bony walls and healthy baseline conditions. Moreover, the observed partial vertical soft tissue rebound highlights the importance of considering ongoing soft tissue remodeling when determining the optimal timing for subsequent restorative interventions, particularly in esthetically demanding regions. This study was conducted in healthy orthodontic patients with intact extraction socket walls, and orthodontic tooth movement was deferred for 90 days after extraction. Accordingly, the present findings reflect early soft tissue healing under favorable baseline conditions. Caution is therefore required when generalizing these results to periodontally compromised patients, sites with pre-existing defects, or esthetic zones, where soft tissue healing patterns may differ.

Limitations of this study include the relatively short 90-day follow-up period and the lack of direct measurements of soft tissue thickness. Although intraoral scanning with superimposition provides reliable surface data, it does not provide information on soft tissue thickness or underlying bone changes. In addition, intra-examiner reliability was assessed using repeated measurements from a subset of sites, which should be considered when interpreting the high ICC values.

Further research, incorporating extended follow-up periods, larger sample sizes, and multimodal evaluation, will be essential to understand post-extraction soft tissue healing better and refine ARP protocols for various clinical situations.

## Conclusions

Within the limitations of this study, no significant differences were observed in soft tissue dimensional changes between sites treated with alveolar ridge preservation (ARP) and those that healed spontaneously. However, consistent patterns of more substantial buccal contour reduction relative to the lingual aspect and greater dimensional changes closer to the gingival margin were observed throughout the study period.

These findings suggest that in healthy orthodontic patients, the early soft tissue benefit of ARP may be limited. Nonetheless, the use of intraoral scanning and digital superimposition enabled reliable assessment of post-extraction soft tissue dynamics and may inform more personalized clinical decision-making.

## Supporting information

S1 File Raw data underlying the findings reported in this study. (XLSX)

S2 File STROBE checklist for observational studies. (DOCX)
