# Supplementary material for: Three-dimensional evaluation of soft tissue contour changes after premolar extraction with and without alveolar ridge preservation: A prospective observational study
Source: PLoS One. 2026 Feb 27;21(2):e0342338. doi: 10.1371/journal.pone.0342338 (PMC12948093; doi:10.1371/journal.pone.0342338)
Supplement: S2 File — (DOCX) [file pone.0342338.s002.docx]

STROBE Statement—checklist of items that should be included in reports of observational studies

|  | Item No. | Recommendation | Page  No. | Relevant text from manuscript |
| --- | --- | --- | --- | --- |
| **Title and abstract** | 1 | (*a*) Indicate the study’s design with a commonly used term in the title or the abstract | 1-3 | Title page and abstract |
|  |  | (*b*) Provide in the abstract an informative and balanced summary of what was done and what was found | 2-3 | Abstract |
| Introduction | | | |  |
| Background/rationale | 2 | Explain the scientific background and rationale for the investigation being reported | 4-5 | Introduction |
| Objectives | 3 | State specific objectives, including any prespecified hypotheses | 5 | Introduction |
| Methods | | | |  |
| Study design | 4 | Present key elements of study design early in the paper | 5-6 | Materials and methods - Patient Selection and Sample Size Calculation, Clinical procedures  Figure 1 |
| Setting | 5 | Describe the setting, locations, and relevant dates, including periods of recruitment, exposure, follow-up, and data collection | 5-6 | Materials and methods |
| Participants | 6 | (*a*) *Cohort study*—Give the eligibility criteria, and the sources and methods of selection of participants. Describe methods of follow-up  *Case-control study*—Give the eligibility criteria, and the sources and methods of case ascertainment and control selection. Give the rationale for the choice of cases and controls  *Cross-sectional study*—Give the eligibility criteria, and the sources and methods of selection of participants | 5-6 | Materials and methods |
|  |  | (*b*) *Cohort study*—For matched studies, give matching criteria and number of exposed and unexposed  *Case-control study*—For matched studies, give matching criteria and the number of controls per case | NA |  |
| Variables | 7 | Clearly define all outcomes, exposures, predictors, potential confounders, and effect modifiers. Give diagnostic criteria, if applicable | 5-6 | Materials and methods  No diagnostic criteria were applicable as this was a dimensional measurement study. |
| Data sources/ measurement | 8* | For each variable of interest, give sources of data and details of methods of assessment (measurement). Describe comparability of assessment methods if there is more than one group | 6-7 | Materials and methods |
| Bias | 9 | Describe any efforts to address potential sources of bias | 6-7 | All surgeries were performed by a single operator. Data superimposition, cross-sectional alignment, and linear measurements were performed by a single calibrated examiner. |
| Study size | 10 | Explain how the study size was arrived at | 6 | Sample size calculations |

Continued on next page

| Quantitative variables | 11 | Explain how quantitative variables were handled in the analyses. If applicable, describe which groupings were chosen and why | 7-9 | Quantitative measurements were reported as mean ± SD. No categorization of continuous variables was performed. |
| --- | --- | --- | --- | --- |
| Statistical methods | 12 | (*a*) Describe all statistical methods, including those used to control for confounding | 7 | Intergroup effects were tested with repeated-measures ANOVA (Bonferroni-adjusted), and within-subject comparisons with Wilcoxon signed-rank tests (p<0.05) |
|  |  | (*b*) Describe any methods used to examine subgroups and interactions | NA | No subgroup or interaction analyses were performed. |
|  |  | (*c*) Explain how missing data were addressed | NA | There were no missing data; all participants completed all follow-up visits. |
|  |  | (*d*) *Cohort study*—If applicable, explain how loss to follow-up was addressed  *Case-control study*—If applicable, explain how matching of cases and controls was addressed  *Cross-sectional study*—If applicable, describe analytical methods taking account of sampling strategy | NA | Not applicable: no loss to follow-up occurred. |
|  |  | (*e*) Describe any sensitivity analyses | NA | No sensitivity analyses were conducted. |
| Results | | | | |
| Participants | 13* | (a) Report numbers of individuals at each stage of study—eg numbers potentially eligible, examined for eligibility, confirmed eligible, included in the study, completing follow-up, and analysed | 7 | 18 patients were assessed for eligibility, enrolled, completed all follow-ups, and included in the final analysis. |
|  |  | (b) Give reasons for non-participation at each stage | NA | Not applicable; no dropouts occurred. |
|  |  | (c) Consider use of a flow diagram | NA | Not included; all participants completed study without exclusions. |
| Descriptive data | 14* | (a) Give characteristics of study participants (eg demographic, clinical, social) and information on exposures and potential confounders | 6 | Healthy orthodontic patients ≥18 years; no other data collected. |
|  |  | (b) Indicate number of participants with missing data for each variable of interest | NA | No missing data. |
|  |  | (c) *Cohort study*—Summarise follow-up time (eg, average and total amount) | 6-7 | All participants completed 90-day follow-up. |
| Outcome data | 15* | *Cohort study*—Report numbers of outcome events or summary measures over time | NA | Not applicable: no events, only dimensional measurements. |
|  |  | *Case-control study—*Report numbers in each exposure category, or summary measures of exposure |  |  |
|  |  | *Cross-sectional study—*Report numbers of outcome events or summary measures |  |  |
| Main results | 16 | (*a*) Give unadjusted estimates and, if applicable, confounder-adjusted estimates and their precision (eg, 95% confidence interval). Make clear which confounders were adjusted for and why they were included | 7-9 | Mean ± SD and percentage volumetric reduction were reported. p-values for group comparisons and time-dependent changes were provided. No confounder adjustment was performed due to homogeneous sample. |
|  |  | (*b*) Report category boundaries when continuous variables were categorized | NA |  |
|  |  | (*c*) If relevant, consider translating estimates of relative risk into absolute risk for a meaningful time period | NA |  |

Continued on next page

| Other analyses | 17 | Report other analyses done—eg analyses of subgroups and interactions, and sensitivity analyses | NA | No subgroup or sensitivity analyses. |
| --- | --- | --- | --- | --- |
| Discussion | | | | |
| Key results | 18 | Summarise key results with reference to study objectives | 10-11 | Discussion: Main findings summarized. |
| Limitations | 19 | Discuss limitations of the study, taking into account sources of potential bias or imprecision. Discuss both direction and magnitude of any potential bias | 11 | Discussion: Sample size, short follow-up, healthy sites and surface data only. |
| Interpretation | 20 | Give a cautious overall interpretation of results considering objectives, limitations, multiplicity of analyses, results from similar studies, and other relevant evidence | 10-11 |  |
| Generalisability | 21 | Discuss the generalisability (external validity) of the study results | 11 | Limited to healthy orthodontic patients, 90-day follow-up. |
| Other information | |  | | |
| Funding | 22 | Give the source of funding and the role of the funders for the present study and, if applicable, for the original study on which the present article is based | entered in the online submission system | Grant no. 08-2019-0003 |

*Give information separately for cases and controls in case-control studies and, if applicable, for exposed and unexposed groups in cohort and cross-sectional studies.

**Note:** An Explanation and Elaboration article discusses each checklist item and gives methodological background and published examples of transparent reporting. The STROBE checklist is best used in conjunction with this article (freely available on the Web sites of PLoS Medicine at http://www.plosmedicine.org/, Annals of Internal Medicine at http://www.annals.org/, and Epidemiology at http://www.epidem.com/). Information on the STROBE Initiative is available at www.strobe-statement.org.
